# Supplementary material for: Warming up human body by nanoporous metallized polyethylene textile
Source: Nat Commun. 2017 Sep 19;8:496. doi: 10.1038/s41467-017-00614-4 (PMC5605506; doi:10.1038/s41467-017-00614-4)
Supplement: Supplementary file 1 — Supplementary Information [file 41467_2017_614_MOESM1_ESM.pdf]

Description of Supplementary Files

File Name: Supplementary Information
Description: Supplementary Figures, Supplementary Tables, Supplementary Note and Supplementary References

File Name: Peer Review File

Supplementary Note 1. Heat transfer model analysis

We used one-dimensional steady-state heat transfer model analysis to determine the impact of textile's IR properties on the required set-point of environment temperature for maintaining thermal comfort in a cold environment. This model includes the heat radiation, conduction and convection to simulate the heat dissipation from a clothed human body to the ambient environment (Supplementary Fig. 1). The criterion for thermal comfort is set as the equality of the total heat dissipation rate with the total heat generation rate. Based on energy balance, the heat dissipation flux should equal the metabolic heat generation flux at every position in the system. The net radiation equations with non-radiative components of heat transfer are as follows (first index refers to inward or outward flow),

At surface s (skin)

$$q_{in,s} + q_{gen} = q_{out,s} + q_{cond,a} \tag{1}$$

$$q_{out,s} = q_{rad,s} + (1 - \varepsilon_s)\, q_{in,s} \tag{2}$$

At surface e (environment)

$$q_{in,e} + q_{conv} = q_{out,e} + q_{gen} \tag{3}$$

$$q_{out,e} = q_{rad,e} + (1 - \varepsilon_e)\, q_{in,e} \tag{4}$$

For the absorbing layer (textile) with inner surface i and outer surface o,

$$q_{out,i} = q_{rad,i} + (1 - \tau_t - \varepsilon_i)\, q_{in,i} + \tau_t\, q_{in,o} \tag{5}$$

$$q_{out,o} = q_{rad,o} + (1 - \tau_t - \varepsilon_o)\, q_{in,o} + \tau_t\, q_{in,i} \tag{6}$$

The outward and inward radiative heat fluxes q_{out} and q_{in} are related as,

$$q_{in,o} = q_{out,e}\,, \quad q_{in,i} = q_{out,s}\,, \quad q_{in,s} = q_{out,i}\,, \quad q_{in,e} = q_{out,o} \tag{7}$$

The energy balance equations at the skin surface and textile outer surface are as follows ($\varepsilon_e = 1$),

At skin surface

$$q_{\mathrm{gen}} = (1 - \rho_{\mathrm{i}})\ [q_{\mathrm{rad,s}} + (1 - \varepsilon_{\mathrm{s}})\ q_{\mathrm{in,s}}] - \tau_{\mathrm{t}}\ q_{\mathrm{rad,e}} - q_{\mathrm{rad,i}} + q_{\mathrm{cond,a}} \tag{8}$$

At textile outer surface

$$q_{\mathrm{gen}} = \tau_{\mathrm{t}}\ [q_{\mathrm{rad,s}} + (1 - \varepsilon_{\mathrm{s}})\ q_{\mathrm{in,s}}] - q_{\mathrm{rad,e}} + \rho_{\mathrm{o}}\ q_{\mathrm{rad,e}} + q_{\mathrm{rad,o}} + q_{\mathrm{conv}} \tag{9}$$

Within the textile, the temperature profile has the following analytical formulation[1],

$$T_{\mathrm{o}} = t_{\mathrm{t}}/(2k_{\mathrm{t}}) \times [q_{\mathrm{rad,i}} + q_{\mathrm{rad,o}} - \alpha_{\mathrm{i}}\ (q_{\mathrm{rad,s}} + (1 - \varepsilon_{\mathrm{s}})\ q_{\mathrm{in,s}}) - \alpha_{\mathrm{o}}\ q_{\mathrm{rad,e}}] - k_{\mathrm{a}}t_{\mathrm{t}}/(k_{\mathrm{t}}t_{\mathrm{a}}) \times (T_{\mathrm{s}} - T_{\mathrm{i}}) + T_{\mathrm{i}} \tag{10}$$

Here, q_{gen} is the metabolic heat generation rate per unit area, $\mathrm{q}_{\mathrm{rad,s}}$ is the radiative heat flux from the skin, $q_{\mathrm{rad,e}}$ is the radiative heat flux from the ambient environment, $q_{\mathrm{rad,i}}$ is the radiative heat flux from the textile inner surface, $q_{\mathrm{rad,o}}$ is the radiative heat flux from the textile outer surface, $q_{\mathrm{cond,a}}$ is the conductive heat flux in the air gap between the skin and the textile, q_{conv} is the convective heat flux from the textile to the ambient environment. According to Fourier's law, Newton's law of cooling and the Stefan-Boltzmann law, the conductive, convective and radiative heat flux terms can be expressed as,

$$q_{\mathrm{rad,s}} = \varepsilon_{\mathrm{s}} \cdot \sigma T_{\mathrm{s}}^4 \tag{11}$$

$$q_{\mathrm{rad,e}} = \varepsilon_{\mathrm{e}} \cdot \sigma T_{\mathrm{e}}^4 \tag{12}$$

$$q_{\mathrm{rad,i}} = \varepsilon_{\mathrm{i}} \cdot \sigma T_{\mathrm{i}}^4 \tag{13}$$

$$q_{\mathrm{rad,o}} = \varepsilon_{\mathrm{o}} \cdot \sigma T_{\mathrm{o}}^4 \tag{14}$$

$$q_{\mathrm{cond,a}} = (k_{\mathrm{a}}/t_{\mathrm{a}}) \cdot (T_{\mathrm{s}} - T_{\mathrm{i}}) \tag{15}$$

$$q_{\mathrm{conv}} = h \cdot (T_{\mathrm{o}} - T_{\mathrm{e}}) \tag{16}$$

All the input parameters are listed in Supplementary Table 1 below.[2-5] Note that the textile thermal conductivity k_{t} is an effective value of the textile as an entirety, in which the existence of air gaps in the textile is considered.[6-9]

Based on the above equations and known parameters, the three unknowns, T_e, T_i and T_o, can be solved with the input of ε_i and ε_o values from 0 to 1. The resulted T_e corresponds to the required set-point for the specific textile to maintain the thermal comfort at the constant skin temperature (33 °C) and heat generation rate (73 W m^{-2}).

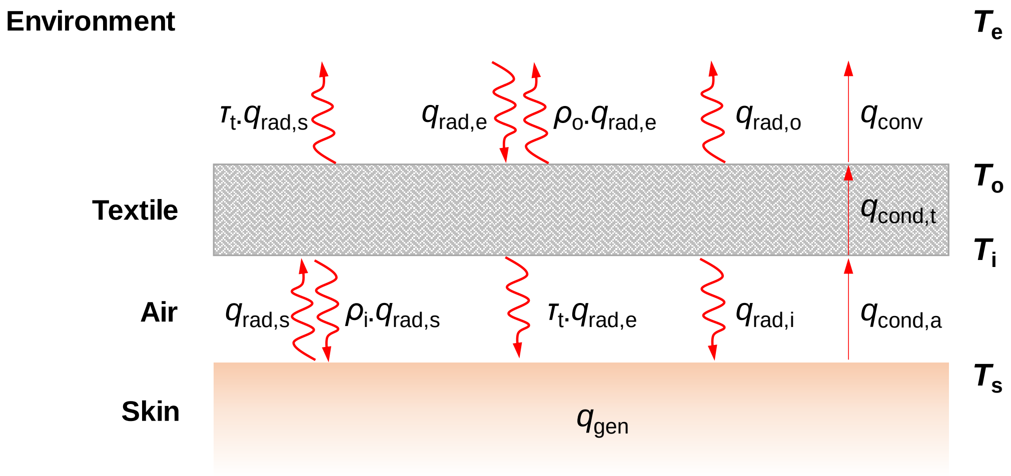

Supplementary Figure 1. Schematic of the heat transfer model. Schematic illustrating the heat transfer model of clothed human body.

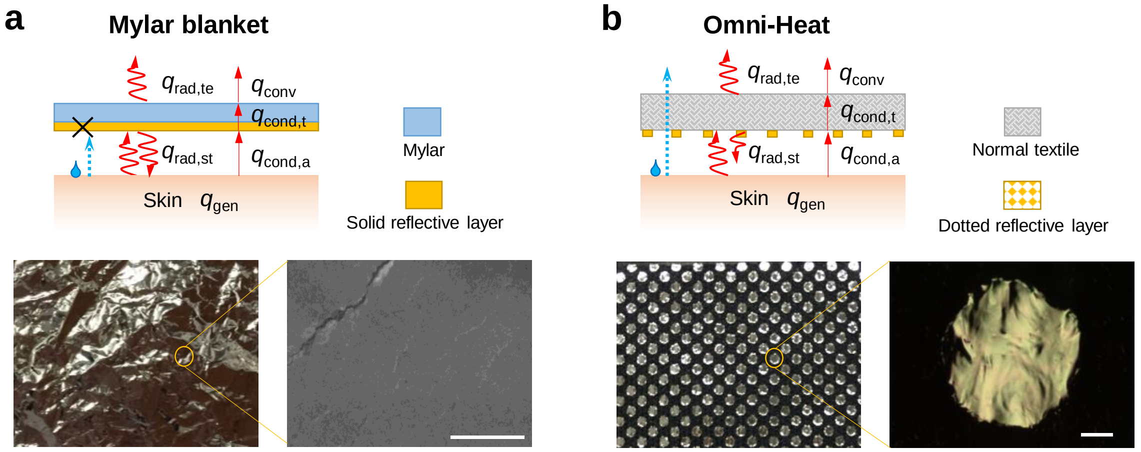

Supplementary Figure 2. Illustration of Mylar blanket and Omni-heat. (a) Schematic of Mylar blanket and its optical and SEM images. Scale bar, 1 μm. (b) Schematic of Omni-Heat and its optical images. Scale bar, 200 μm.

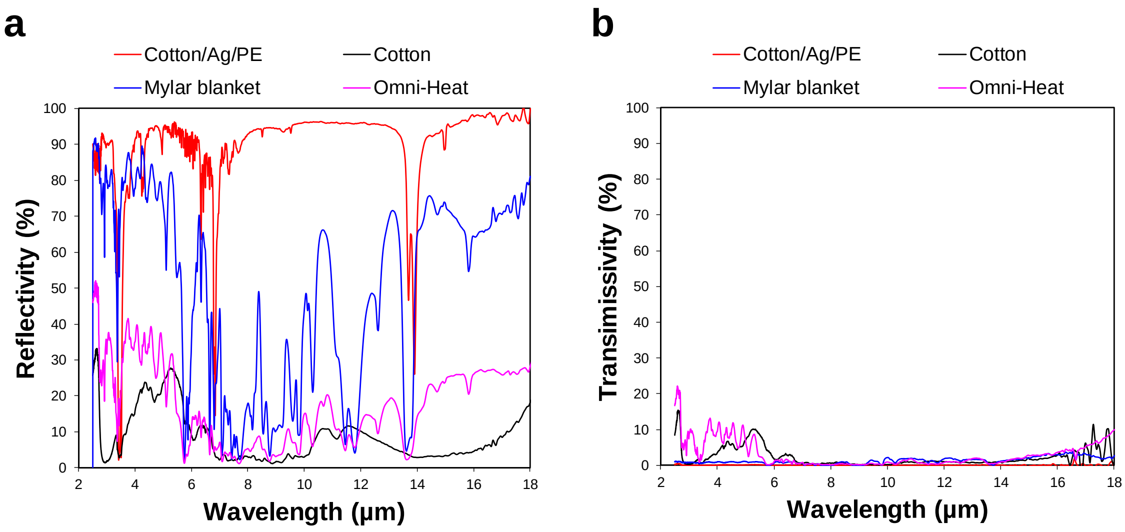

Supplementary Figure 3. Additional characterization of infrared property. (a) IR reflectance and (b) transmittance spectra of cotton/Ag/PE, cotton, Mylar blanket and Omni-Heat.

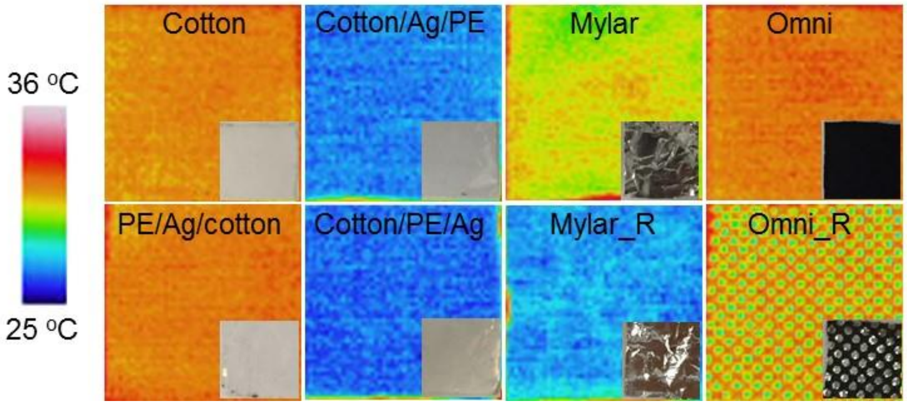

Supplementary Figure 4. Additional thermal characterization. Thermal imaging and photos of the textile samples covering the simulated skin.

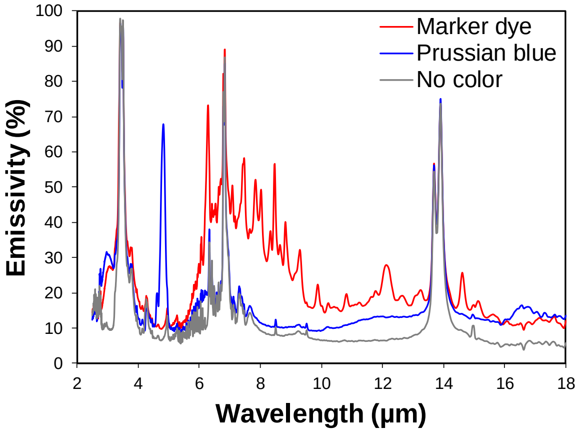

Supplementary Figure 5. Coloration effect on IR emissivity. IR emissivity spectra of cotton/Ag/PE textiles before and after coloration with Prussian blue and permanent marker dye.

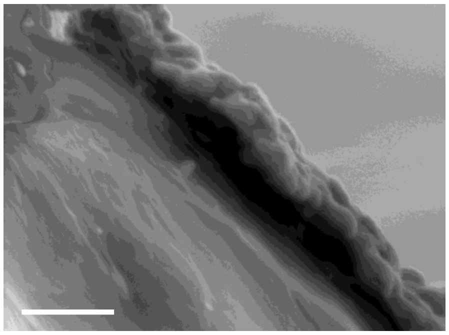

Supplementary Figure 6. Thickness of the Ag layer. SEM image of the cross-sectional view of electrolessly plated Ag film. Scale bar, 500 nm.

Supplementary Table 1. Input parameters for the heat transfer model analysis.

Symbol	Definition	Value	Unit
q_{gen}	Metabolic heat generation flux	73	W m^{-2}
k	Thermal conductivity	Textile, k_t = 0.05<br>Air gap, k_a = 0.03	W m^{-1} K^{-1}
t	Thickness	Textile, t_t = 350<br>Air gap, t_a = 1400	μm
h	Natural convective heat transfer coefficient	4.1	W m^{-2} K^{-1}
σ	Stefan-Boltzmann constant	5.67×10^{-8}	W m^{-2} K^{-4}
ε	IR emittance	Skin, ε_s = 0.98<br>Environment, ε_e = 1<br>Cotton, ε_c = 0.89<br>Textile inner surface, ε_i<br>Textile outer surface, ε_o	unitless
α	IR absorbance	Cotton, α_c = 0.89<br>Textile inner surface, $\alpha_i = \varepsilon_i$<br>Textile outer surface, $\alpha_o = \varepsilon_o$	unitless
τ	IR transmittance	Cotton, τ_c = 0.02<br>Opaque textile, τ_t = 0	unitless
ρ	IR reflectance	Cotton, ρ_c = 0.09<br>Textile inner surface, $\rho_i = 1 - \tau_t - \varepsilon_i$<br>Textile outer surface, $\rho_o = 1 - \tau_t – \varepsilon_o$	unitless
T	Temperature	Skin, T_s = 306.15<br>Environment temperature, T_e<br>Textile inner surface, T_i<br>Textile outer surface, T_o	K

Supplementary Table 2. Comparison of IR properties of normal textile, nanoPE (for cooling), and nanoporous metallized polyethylene (for warming).

	IR transmissivity τ (%)	IR reflectivity ρ (%)	IR emissivity ε (%)
Normal textile (*e.g.*, cotton)	2	9	89
NanoPE	96	3	1
Nanoporous metallized polyethylene	0	90	10

Supplementary References:

1 Tong, J. K. *et al.* Infrared-Transparent Visible-Opaque Fabrics for Wearable Personal Thermal Management. *ACS Photonics* **2**, 769-778 (2015).

2 Mayor, T. S., Couto, S., Psikuta, A. & Rossi, R. M. Advanced modelling of the transport phenomena across horizontal clothing microclimates with natural convection. *International Journal of Biometeorology* **59**, 1875-1889 (2015).

3 Baxter, S. The thermal conductivity of textiles. *Proceedings of the Physical Society* **58**, 105 (1946).

4 Steketee, J. Spectral emissivity of skin and pericardium. *Physics in Medicine and Biology* **18**, 686 (1973).

5 American Society of Heating, R. & Engineers, A.-C. *2009 ASHRAE Handbook: Fundamentals*. (American Society of Heating, Refrigeration and Air-Conditioning Engineers, 2009).

6 Zhu, F. & Li, K. Determining Effective Thermal Conductivity of Fabrics by Using Fractal Method. International Journal of Thermophysics 31, 612-619 (2010).

7 Majumdar, A., Mukhopadhyay, S. & Yadav, R. Thermal properties of knitted fabrics made from cotton and regenerated bamboo cellulosic fibres. International Journal of Thermal Sciences 49, 2042-2048 (2010).

8 Oğlakcioğlu, N. & Marmarali, A. Thermal comfort properties of some knitted structures. Fibres & Textiles in Eastern Europe 15, 64-65 (2007).

9 Yamashita, Y., Yamada, H. & Miyake, H. Effective Thermal Conductivity of Plain Weave Fabric and its Composite Material Made from High Strength Fibers. Journal of Textile Engineering 54, 111-119 (2008).
